# A Prospective Study Investigating Immune Checkpoint Molecule and CD39 Expression on Peripheral Blood Cells for the Prognostication of COVID-19 Severity and Mortality

**DOI:** 10.3390/v16050810

**Published:** 2024-05-20

**Authors:** Thilo Gambichler, Jonas Rüth, Silke Goesmann, Stefan Höxtermann, Marina Skrygan, Laura Susok, Jürgen C. Becker, Oliver Overheu, Wolfgang Schmidt, Anke Reinacher-Schick

**Affiliations:** 1 Department of Dermatology, Ruhr-University Bochum, 44791 Bochum, Germany; 2 Department of Dermatology, Hospital Dortmund, Faculty of Health/School of Medicine, Witten-Herdecke University, 44137 Dortmund, Germany; 3 Department of Dermatology, Christian Hospital Unna, 59423 Unna, Germany; 4 Translational Skin Cancer Research, DKTK Partner Site Essen/Düsseldorf, West German Cancer Center, Dermatology, University Duisburg-Essen, 45122 Essen, Germany; 5 German Cancer Research Center (DKFZ), 69120 Heidelberg, Germany; 6 Department for Internal Medicine, Ruhr-University Bochum, 44791 Bochum, Germany; 7 Department for Hematology and Onoclogy with Palliative Care Unit, Ruhr-University Bochum, 44791 Bochum, Germany

**Keywords:** SARS-CoV-2, COVID-19, flow cytometry, lymphocytes, biomarkers, comorbidities

## Abstract

In patients with COVID-19, broad panels of immune checkpoint molecules (ICPMs) and the purinergic signaling have not been studied in parallel. We aimed to perform in-depth immunophenotyping of major cell subsets present in human peripheral blood of COVID-19 patients and controls using PD1, TIM3, LAG3, TIGIT, and CD200R, as well as CD39, as markers for the purinergic signaling pathway. We studied 76 COVID-19 patients and 12 healthy controls using peripheral blood mononuclear cells on flow cytometry. Univariable and multivariable statistics were performed. All ICPMs studied were significantly overexpressed on different cell subsets of COVID-19 patients when compared with healthy controls. Elevated lactate dehydrogenase; C-reactive protein; age; and high expression of CD45+, CD39+CD45+, TIM3+CD39+CD4+CD45+, and TIM3+CD39+CD8+CD3+CD4+ cells were significantly associated with severe COVID-19. On multivariable analysis, however, only high expression of CD39+CD45+ (OR 51.4, 95% CI 1.5 to 1763) and TIM3+CD39+CD4+CD3+CD45+ (OR 22.6, 95% CI 1.8 to 277) cells was an independent predictor for severe COVID-19. In conclusion, numerous ICPMs are overexpressed in COVID-19 patients when compared with healthy controls, suggesting a pathophysiological role of these molecules in SARS-CoV-2 infection. However, only TIM3 in co-expression with CD39 remained as a significant independent prognostic ICPM on multivariable analysis. The flow cytometric evaluation of TIM3+CD39+CD4+CD3+CD45+, as well as CD39+CD45+, is a powerful tool for the prognostication of COVID-19 patients on hospital admission.

## 1. Introduction

On May 2023, the head of the World Health Organization (WHO) has declared an end to coronavirus disease 2019 (COVID-19) as a public health emergency [1]. Nevertheless, there is still a high need for prognostic markers for risk stratification and optimizing hospitalization admission and monitoring of COVID-19 patients [2,3,4]. Indeed, laboratory parameters may provide prognostic information, which can have a strong impact on the triage, care, and mortality of the patient. Clinical features, including age and comorbidities (e.g., lung disorders, cardiovascular diseases, and diabetes mellitus) and easily available laboratory parameters (e.g., C-reactive protein (CRP), lactate dehydrogenase (LDH), lymphocyte count, and absolute eosinopenia), have been established as consistent predictors of severe COVID-19 (e.g., pneumonia or immediate care unit admission) and a fatal outcome [2,3,4,5]. 

However, more in-depth profiling of blood cell subpopulations and immune checkpoint molecules (ICPMs) may even improve COVID-19 prognostication and contribute to a better understanding of COVID-19 immunopathogenesis [6].

The exhaustion of T cells is one of the most important factors leading to decreased T-cell activity against malignancies, as well as infectious agents. Importantly, the exhaustion of T cells is a characteristic finding in most chronic viral infections, including HIV and hepatitis B virus infection. Exhausted T lymphocytes are functionally characterized by loss of IL-2 production, impaired proliferation, diminished cytotoxicity, and altered production of proinflammatory cytokines. During some acute viral infections (e.g., flu or COVID-19), PD1 as well as other ICPMs, such as LAG3, TIM3, TIGIT, and CD200, are up-regulated [7,8,9,10,11,12,13,14,15]. However, the pathogenesis of acute viral infections differs from that of chronic infections in that the immune system is particularly activated by viral particles in the acute phase. At the end of the infection, after the virus has been eliminated, memory T cells differentiate [16]. After viral infection, naive CD8+ T cells upregulate PD-1 even before their cell division is complete, and the transition to effector T cells is inhibited by the PD-1/PD-L1 pathway at early stages of infection [17]. This suggests that PD-1 prevents excessive immunopathology at early stages [17,18]. On the other hand, both hematopoietic and non-hematopoietic cells express increased levels of PD-L1 and PD-L2 ligands following viral infection, which can lead to viral immune evasion by depleting and suppressing the viral immune response of T cells [16,18]. Persistent and excessive expression of PD-1 in the early phase of acute infections can enable the virus to evade the immune system and counteract severe inflammatory reactions triggered by the infection, potentially leading to severe, fatal, or chronic courses of infectious diseases. [7,8,9,10,11,12,13,14,15,16,18]. For example, in acute viral infections, the function of TIM-3 differs from that of PD-1. On one side, the expression of TIM-3 on T cells promotes the development of short-lived effector T cells into an effective T-cell response through costimulation; however, TIM-3 also causes suppression of the adaptive immune response by restricting the development of virus-specific memory T cells in the acute phase [19]. 

Similar to antigen-presenting cells, CD4+ T cells mediate effector function in the early phase of inflammation. In SARS-CoV-2, CD4+ was shown to be dominant in spike-specific T-cell function, irrespective of the viral variant [20]. Helper T cells are also involved in the adaptive immune response by developing into memory T cells. Thus, a correlation of follicular SARS-CoV-2-specific T cells with humoral immunity has been demonstrated [20,21,22]. Effective control of COVID-19 is associated with a type 1 CD4 phenotype, whereas type 2 is more common in patients with severe disease [23]. Furthermore, for the SARS-CoV-2-specific T-cell memory response, it has been shown that CD4+ memory T cells are more common than CD8+ T cells, are characterized by a polyfunctional profile with increased IL-2 and decreased IFN-y secretion, and tend to have less cytotoxic function [20]. They also have a central memory profile, which is why high levels of them are associated with prolonged immunity [20,24]. 

During the last four years, many papers have addressed inhibitory immune checkpoint receptors and ligands as prognostic biomarkers in COVID-19 patients. Overall, the overexpression of ICPMs in the early phase of infection might be useful as prognostic biomarkers for COVID-19 severity/fatality [7,8,9,10,11,12,13,14,15]. In previous studies, however, the purinergic signaling pathway has not been studied paralleled with ICPMs. Indeed, the expression of CD39 is an indicator of T-cell exhaustion as well and might be a potent biomarker for the prognostication of COVID-19 severity [20,21]. All in all, however, the data on CD39 expression in the context of COVID-19 are relatively sparse, and there are no studies simultaneously investigating the purinergic signaling pathway, as well as a broader panel of ICPMs in COVID-19 patients in order to find independent prognostic biomarkers under the exclusion of other confounding laboratory and clinical parameters [22,23,24,25,26,27,28,29,30].

To detect significant independent baseline biomarkers for COVID-19 severity, we aimed to perform in-depth immunophenotyping of major cell subsets present in human peripheral blood of COVID-19 patients and controls using a panel of ICPMs (PD1, TIM3, LAG3, TIGIT, and CD200R) and CD39 as markers for the purinergic signaling pathway.

## 2. Materials and Methods

### 2.1. Patients

This prospective study recruited COVID-19 patients at a tertiary care hospital (St. Josef) of the Ruhr-University Bochum (Bochum, Germany). Laboratory-confirmed COVID-19 patients were included in the study who had a blood collection on admission. The SARS-CoV-2 detection was carried out using nasopharyngeal swab specimens and a commercial qPCR assay (AllplexTM 2019-nCoV, Seegene, Republic of Korea) according to a standard protocol [31]. In addition, we aimed to study samples of COVID-19 patients with an increased risk profile for more complicated courses. The number of cases in the patient group was estimated according to the regional and national course of the COVID-19 waves; the number of moderate, severe, and fatal cases included; and the reproducibility of the test results. Patients were continuously recruited over a period of 14 months, focusing on patients from infectious and intensive care units. Pregnant females; children (age < 18 years); those with other conditions affecting laboratory parameters, including those with chronic hematological conditions and corticosteroid medication on admission; and those whose outcomes were unknown were excluded from this investigation. Moreover, we recruited matched healthy controls after data collection in the COVID-19 group. For this purpose, inpatients with a negative COVID-19 test who had an increased risk profile for severe COVID-19 and more than two relevant previous illnesses were selected. The selection was also made with the aim of achieving a matched sex and age distribution, focusing on the median age.

### 2.2. Data Extraction and Outcome Measure

At first contact, patients were asked about their individual risk profiles, previous illnesses, vaccination statuses, and previous infections, and vital parameters were determined by a medical doctor and a trained medical student. The patients were then followed throughout their hospital stay through regular review of their electronic medical files by the research team. These data included patients’ characteristics, comorbidities, length of in-patient treatment, treatment details, laboratory data, and the clinical outcomes. More details are given in Table 1. COVID-19 progression and the final disease outcome of COVID-19 were evaluated by the WHO clinical progression scale [32]: I, score 1–3 (ambulatory mild disease); II, score 4 and 5 (hospitalized: moderate disease); III, score 6–9 (hospitalized: severe disease); and IV, score 10 (death). For statistical analysis, we dichotomized the WHO clinical progression scale in class I and II (0) vs. class III and IV (1). 

### 2.3. Peripheral Blood Mononuclear Cell Isolation

In this study, 8 mL of heparinized venous blood was collected from each participant. Peripheral mononuclear lymphocytes (PBMCs) were isolated using the BD Vacutainer^®^ (Franklin Lakes, NJ, USA) CPTTM mononuclear cell preparation tube system with integrated FICOLLTM gradient. After 20 min of vertexation at 1650 g, the cell suspensions were removed from the CPT tube and transferred to a 50 mL conical tube. The volume was filled to 50 mL using RPMI 1640 medium. The samples were then centrifuged at 250 g at room temperature for 7 min. After centrifugation, the supernatant was removed and discarded. In order to preserve the obtained PBMCs, they were stored after incubation with a freezing medium of 90% FBS/10% DMSO and at a temperature of −80 °C.

### 2.4. Flow Cytometry

**Figure 1:**
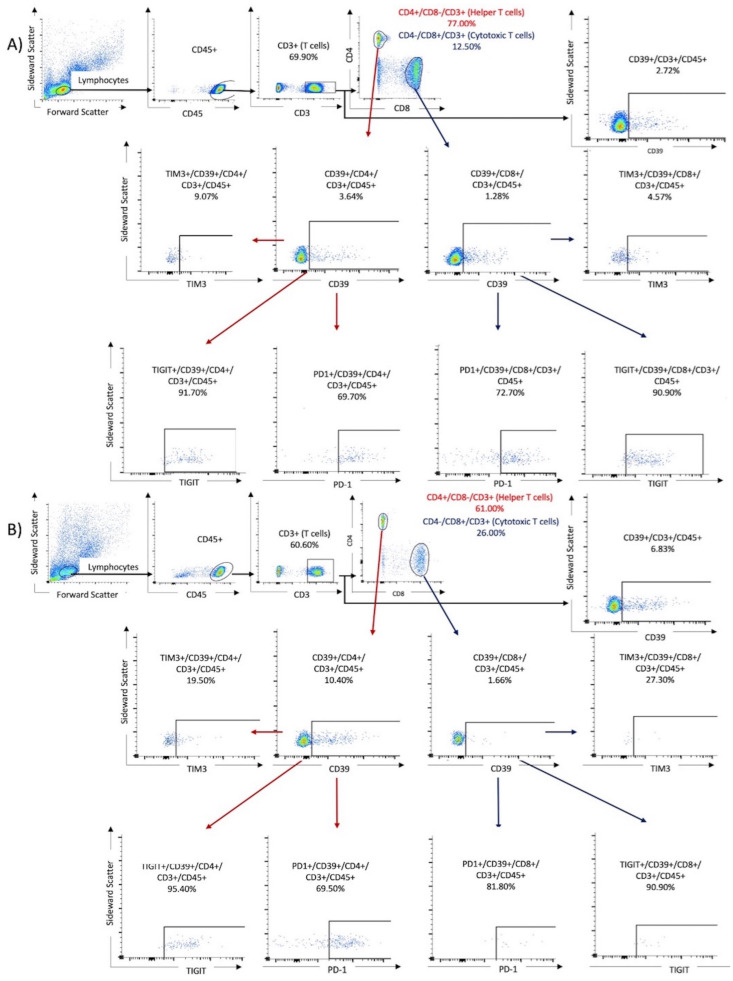
The gating algorithm for the expression of CD39 and immune checkpoint molecules on T cells. Results of two samples were shown: (**A**) a 76-year-old male from the non-critical COVID group (WHO I and II) and (**B**) an 80-year-old male from the critical COVID-19 group (WHO III and IV).

To perform flow cytometry, the frozen PBMC samples were thawed, and a cell suspension was prepared. First, 1 mL of preheated RPMI was added dropwise to the cooled samples. The mixture was then centrifuged at 250 g at room temperature. Next, another 25 mL of RPMI was added to the mixture to remove and discard the supernatant after the second centrifugation at 250 g. Finally, the cells were reperfused with 400 μL of BD dye buffer. For the analysis, PBMCs were stained with the following BD OptiBuild^TM^ (Franklin Lakes, NJ, USA) fluorochrome mouse anti-human monoclonal antibodies (Franklin Lakes, NJ, USA) listed in Table 2. To prepare the compensation mixture for the FACS assay, 100 μL of the cell suspension was mixed with 5 μL of the respective antibody and incubated in the dark for 30 min at room temperature. After two washes, the cell samples underwent analysis using the BD FACSCelesta (Franklin Lakes, NJ, USA) and were identified by their antibody labels after cell sorting (refer to Table 2). The resulting data were analyzed using FACSDiva software version 9.0 (Franklin Lakes, NJ, USA) and FlowJo software version 10.8.1 1 (Ashland, OR, USA). Lymphocytes were identified by their forward and lateral scattering and verified by their CD45+ expression. The analysis was performed for the following three tubes: Tube 1 contained T cells identified by their CD3+/CD4+ or CD3+/CD8+ expression. Tube 2 contained NK cells, first identified by the expression of CD16+ and CD56+, followed by investigation of ICPMs on these cells. Tube 1 was also used to investigate the expression of CD39 and the co-expression of CD39 and ICPMs. Tube 3 was used to observe the expression of CD200R1 on CD11c+, CD14+, CD16+, and CD66a+ expressing monocytes. The subpopulations were gated by comparison with antibody-free negative controls (fluorescence minus one). The cutoff limit was defined as the maximum expression in the antibody-negative controls. Cell populations beyond this limit were considered as positivity gates. Figure 1 displays the gating algorithm for the co-expression of ICMPs and CD39 on T cells (Tube 1) in samples from a patient with mild disease and one with severe COVID-19.

### 2.5. Statistics

The MedCalc (Ostende, Belgium) software version 20.009 was used. Analysis of data distribution was performed by the D’Agostino–Pearson test. Univariable statistics included the Chi^2^ test for dichotomized data and receiver operating characteristics (ROC) analyses for continuous data [including associated criterion, area under the curve (AUC), and Youden index (optimal cutoff points of both the maximum sensitivity and specificity)]. Continuous data were dichotomized as dummy variables according to the associated criterion obtained from ROC analyses. Multivariable analysis was performed using a logistic regression model, exclusively including data obtained from univariable testing if (1) significant with an AUC of ≥0.70 on ROC analysis or (2) significant on Chi^2^ analysis using categorial data. Odds ratios (OR) including the 95% confidence intervals (CI) were calculated as well. As required, the independent variables did not strongly correlate with each other. In order to evaluate the logistic regression model, we also used ROC curve analysis. *p* < 0.05 was considered statistically significant. 

## 3. Results

### 3.1. Patient Characteristics

We studied 76 mRNA-confirmed COVID-19 patients [median age: 64 years (22–87); 47 males (61.%), 29 females (38.2%)] and 12 healthy controls [median age: 63 years (47–73); 8 males (66.7%), 4 females (33.3%)]. With respect to sex (*p* = 0.99) and age (*p* = 0.61), there was no significant difference between COVID-19 patients and healthy controls. Further details of the clinical characteristics of the investigated COVID-19 patients are listed in Table 1. According to the WHO clinical progression scale we observed 9/76 (11.8%) with severe disease including 3/76 (3.9%) with fatal outcomes. In this cohort, 13 patients required treatment in the intensive care unit (ICU) with a median stay of 17 days (17.1%, range 1–82). 

### 3.2. Flow Cytometry

**Figure 2:**
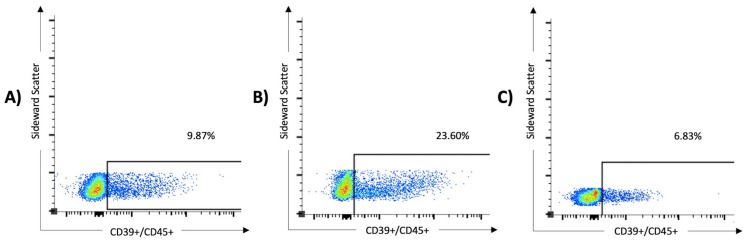
The expression of CD39 on lymphocytes was significantly higher in patients with severe SARS-CoV-2 infection than in those with mild disease and healthy controls. In the multivariate analysis, overexpression of CD39+/CD45+ serves as a predictor of severe COVID-19 (odds ratio 51.4, 95% CI 1.5 to 1763). The results of three samples are shown: (**A**) a 76-year-old male from the non-critical COVID group (WHO I and II, 9.87% of the lymphocytes express CD39), (**B**) an 80-year-old male from the critical COVID group (WHO III and IV, 23.6% of the lymphocytes express CD39), and (**C**) a 63-year-old female from the control group (6.83% of the lymphocytes express CD39).

The solo expression and coexpression of ICPMs and CD39 on T cells was higher in cases with severe COVID-19 disease than in mild cases, as illustrated in Figure 1. As shown in Table 3, CD8+CD3+CD45+ (*p* = 0.011), CD56+CD45+ (*p* < 0.0001), and CD56+CD15+CD45+ (*p* < 0.0001) cells were significantly decreased in COVID-19 patients when compared with healthy controls. By contrast, CD39+CD3+CD45+ (*p* = 0.0004), CD39+CD4+CD3+CD45+ (*p* = 0.016), and CD39+CD8+CD3+CD45+ (*p* = 0.0089) cells were significantly increased in COVID-19 patients. Figure 2 demonstrates the increased proportion of CD39+ expressing cells in lymphocytes, identified by its scattering properties and CD45 positivity, in severe courses of COVID-19. CD39 overexpression was particularly observed in patients with severe COVID-19. In conclusion, the presented results in Table 3 and Figure 2 indicate that the CD39 signaling pathway may be upregulated, and lymphocyte function may be impaired in the early phase of acute viral infection, leading to T-cell exhaustion and increased complications of COVID-19. Furthermore, a depletion of immune cells was shown under SARS-CoV-2 infection compared with healthy controls.

As demonstrated in Table 4, PD1, TIM3, and LAG3 expression was significantly increased in all cell subsets of COVID-19 patients when compared with healthy controls, except for TIM3+CD4+CD3+CD45+ (*p* = 0.64), TIM3+CD8+CD3+CD45+ (*p* = 0.41), and TIM3+CD39+CD8+CD3+CD45+ (*p* = 0.060). Figures S1–S5 visually represent these results of the FACS analyses. As shown in Figure S6 and Table 5, the relative proportion of monocytes, macrophages, and NK cells expressing CD200R was significantly higher in COVID-19 patients than in healthy controls, suggesting the involvement of this immune checkpoint in the immunological response to COVID-19 infection of these immune cells. Moreover, TIGIT+CD16+CD45+ (*p* < 0.0001), TIGIT+CD56+CD45+ (*p* = 0009), and TIGIT+CD39+CD4+CD3+CD45+ (*p* = 0.0017) cells were significantly increased in COVID-19 when compared with healthy controls. The results indicated no significant difference in antibody expression between female and male subjects in the target gates.

### 3.3. Univariable and Multivariable Analysis

**Figure 3:**
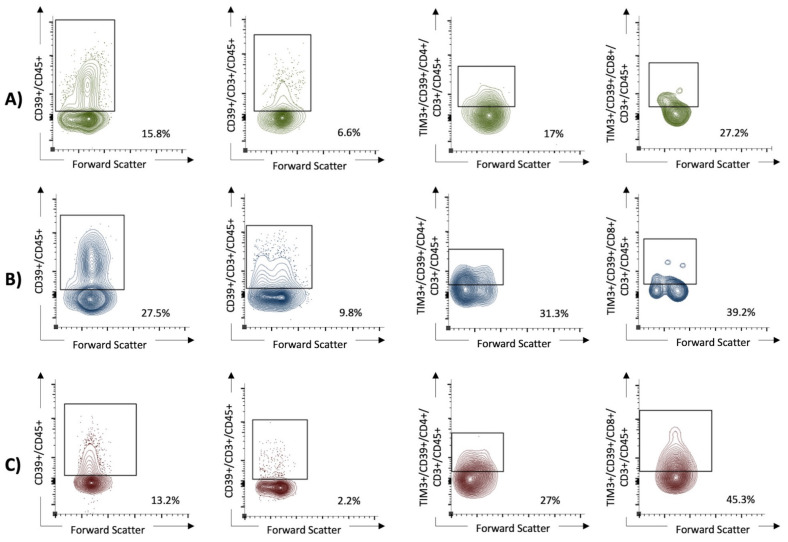
The expression of CD39+ on lymphocytes and the coexpression of TIM3+ and CD39+ on T helper cells (CD4+ T cells), as well as cytotoxic T cells (CD8+ T cells), were in univariable analysis significantly higher in patients with severe SARS-CoV-2 infection than in those with mild disease and healthy controls. In the multivariable analysis, high expression of CD39+CD45+ (OR 51.4, 95% CI 1.5 to 1763) and TIM3+CD39+CD4+CD3+CD45+ (OR 22.6, 95% CI 1.8 to 277) cells remained as an independent predictor for severe COVID-19. (**A**) The non-critical COVID group (WHO I and II). (**B**) The critical COVID group (WHO III and IV). (**C**) The healthy control group.

Univariable analysis revealed that elevated LDH; CRP; age; and high expression of CD39+CD45+, TIM3+CD39+CD4+CD45+, and TIM3+CD39+CD8+CD3+CD4+ cells were significantly associated with severe COVID-19 according to the WHO clinical progression scale (Table 6). Figure 3 shows the increased co-expression of ICPMs TIM3 and CD39 on both CD4+ and CD8+ in patients with severe COVID-19. On multivariable analysis, however, only high expression of CD39+CD45+ (OR 51.4, 95% CI 1.5 to 1763) and TIM3+CD39+CD4+CD3+CD45+ (OR 22.6, 95% CI 1.8 to 277) cells was an independent predictor for severe COVID-19 including the cases with fatal outcomes. An ROC analysis of the logistic regression model revealed a strong discriminating power as indicated by an AUC of 0.93.

## 4. Discussion

In a recent review by Al-Mterin et al. [9], numerous ICPMs (e.g., CTLA-4, BTLA, TIM3, VISTA, LAG3, TIGIT, PD1, Galectin-9, PD-L1, and CD112) were reviewed with regard to their capacity to predict the course of COVID-19. Other authors have also proposed CD200, IDO, and CD223 in this context [7,14]. All these immune checkpoint receptors and ligands have been demonstrated to be upregulated in COVID-19 patients, and some of these appear to have prognostic capacity in this disease. However, most available investigations studied ICPMs individually or only in small combinations [9,10,11]. Indeed, different T-cell inhibitory receptors may be co-expressed during exhausted T-cell differentiation. Hence, Al-Mterin et al. [10] recently reported that comprehensive co-expression and crosstalk studies of multiple ICPMs on specific immune cell subpopulations in COVID-19 patients are lacking. Based on these studies, Al-Mterin et al. [10] suggested that there is some evidence supporting the use of a panel of ICPMs as prognostic biomarkers (e.g., PD1, CTLA-4, TIM3, PD-L1, Gal-3, and Gal-9) for severe COVID-19 patients. In accordance with previous reports [9,10,11], we showed that all studied ICPMs were significantly overexpressed on different cell subsets of COVID-19 patients when compared with healthy controls, indicating that ICPM expression is of pathogenetic significance, and it might be reasonable to use several ICPMs in order to optimize the prognostication of COVID-19 severity.

In a mouse model of acute lymphocyctic choriomeningitis virus infection, Avery et al. [19] demonstrated the involvement of TIM-3 in the increased development of short-lived effector T cells at the expense of progenitor memory T cells. This suggests that TIM-3 acts as a costimulator in the short-term, optimal T-cell response during the early phase of acute viral infection [19]. However, TIM-3 may also mediate the exhaustion of the adaptive immune response by limiting the development of memory T cells in the acute phase. This could lead to severe or chronic disease, which may have prognostic implications [19]. The functions of TIM-3 and PD-1 in acute viral infections are therefore not equivalent. Nevertheless, their expression as markers of potential exhaustion is associated with the progression of COVID-19 in severe disease [20,25]. This does not directly reflect functional exhaustion of T cells but rather a sign of persistent, excessive activation [20,26]. 

In the present study, we investigated a reasonable number of ICPMs and also included CD39 as a marker for the purinergic signaling pathway. CD39 possesses ecto-(Ca^+2^-MG^+2^) apyrase activity enabling hydrolyzation of ATP and ADP into AMP [27,28,33]. The binding of ATP to purinergic receptors activates the purinergic signaling pathway, which is stimulated by the infection. ATP is degraded via CD39 into AMP that is further degraded into adenosine by the ecto-5′-nucleotidase enzyme of CD73. Indeed, CD39 expression significantly affects the function of different immune cells, including CD8+ T cells, which is the most effective cell subset against virus infections. The interaction of CD8+ T cells with extracellular ATP is crucial for enhanced degranulation and cytotoxicity. CD39 represents a marker of T-cell exhaustion that is predominantly observed in chronic viral infections, such as HIV and hepatitis B. The expression of CD39 on CD4+ T cells leads to a higher tendency to apoptosis; lower function of Fox+ regulatory T cells; and, by reducing the release of IL-21, also a lower activation of B cells, which limits T-cell functions in the acute phase of viral infections [29]. Furthermore, CD39 is also expressed on NK cells, and NKT cells regulate macrophage and monocyte activity and chemotaxis [28,30,34,35,36]. 

In COVID-19 patients, there are alterations in the purinergic signaling pathways. During COVID-19 progression, the alteration of CD39/CD73 was reported by Dorneles et al. [37] and correlated with disease severity. The frequency of CD4+CD25−CD39+ T cells was higher in severe cases than in mild cases and healthy controls [38]. Moreover, Pietrobon et al. [39] observed that the percentage of CD39+ cells in CD4+ and CD8+ T cells was higher in patients with severe COVID-19 than in patients with mild infection and healthy controls. Similar results were also reported by Shahbaz et al. [38] and Simsek et al. [40]. However, the aforementioned researchers did not adjust for potential confounders using multivariable analyses. Using a bivariate logistic regression model, however, Diaz-Garcia et al. [41] showed that the plasma concentration of soluble CD39 was increased in patients with COVID-19 and was significantly associated with the duration of hospitalization. Moreover, COVID-19 patients had increased expression of CD39 in CD4+ and CD8+ cells, NK cells, T regulatory cells, and monocytes [33]. 

The aforementioned studies revealed abnormalities in purine metabolism and altered CD39 expression in immune cells during COVID-19 disease. However, CD39 co-expression with different ICPMs was not assessed in these studies. In the present study, we not only showed that a variety of ICPMs were overexpressed when compared with healthy controls but also demonstrated that CD39 expression was significantly upregulated in the T lymphocytes of COVID-19 patients. Notably, Shahbazi et al. [42], who adjusted linear mixed effects models for potential clinical confounders (e.g., age, sex, and comorbidities), observed that critically ill COVID-19 patients had higher frequencies of TIM3+CD8+ and TIM3+CD39+CD8+ cells than non-critical patients. This is a finding that we confirmed on univariable analysis showing that TIM3+CD39+CD8+CD3+CD45+ cells were associated with class III and IV of the WHO clinical progression scale. However, this lymphocyte subset did not remain significant in our logistic regression model. By contrast, we detected that CD39+CD45+ and TIM3+CD39+CD4+CD3+CD45+ were significant independent predictors for severe COVID-19. Accordingly, Modabber et al. [30] reported that TIM3+CD39+CD4+ expression was significantly higher in patients with critical COVID-19 when compared with patients with moderate/severe disease. With respect to the effect size, however, CD39+CD45+ cells appeared to be a stronger predictor for COVID-19 severity as compared with TIM3+CD39+CD4+CD3+CD45+ lymphocytes, as indicated by an OR of 51.4 (95% CI 1.5 to 173) versus an OR of 22.6 (95% CI 1.8 to 277). The wide CIs likely reflected the relatively small investigated sample size. Hence, the evaluation of CD39 expression on numerous hematopoietic cells, except for erythrocytes and plasma cells, is a very useful baseline predictor for COVID-19 severity. Similarly, da Silva et al. [43] showed that moderate and severe cases showed increased expression of CD39 in the total leukocytes of patients with COVID-19.

### Strengths and Limitations

The limitations of this study are evident in the small number of subjects and controls. However, the comparability of the age and sex groups of the two groups is an essential strength that should be emphasized. Furthermore, the number of samples collected from critical stages of the disease was small, but the results were reproducible between samples in both the critical and the non-critical groups. This study was also the first to examine the coexpression of CD39 and ICPMs. In particular, it examined the coexpression of CD39 and TIM-3 with the clinical course of COVID-19. However, it should be noted that the number of lymphocytes in the final cell populations obtained after several gating steps was only a small proportion of the baseline cell population.

## 5. Conclusions

In conclusion, we showed that numerous ICPMs were overexpressed in COVID-19 patients when compared with healthy controls. Hence, we speculated that these ICPMs may play pathophysiological roles in SARS-CoV-2 infection. Adjusting for potential confounders, however, only TIM-3 in co-expression with CD39 remained as significant prognostic ICPMs on multivariable analysis. The co-expression of CD39 and TIM-3 on cell lines of activated T cells was a conceivable predictive marker due to the mediated functions, which could predict T-cell depletion and a severe course with a possible fatal outcome. This was suggested by our results and those published by other groups. The concomitant and excessive upregulation of CD4+ T cells in the early symptomatic phase of COVID-19 may potentially indicate reduced clearance by the immune system via viral immune evasion and reduced activation of Tregs and B cells, as well as possible T-cell exhaustion (20–24). Therefore, the evaluation of TIM3+CD39+CD4+CD3+CD45+, as well as CD39+CD45+, has high power for the prognostication of COVID-19 patients on hospital admission.

## Figures and Tables

**Table 1:** Descriptive clinical baseline data and outcomes of COVID-19 in-patients (n = 76) treated in a German tertiary care hospital.

Parameter	Data
**Sex**	
COVID-19 patients	47 m/29 f (61.8%/38.2%)
Healthy controls	8 m/4 f (66.7%/33.3%); *p* = 0.99
**Median (range) age**	
COVID-19 patients	64 years (22–87)
Healthy controls	63 years (47–73); *p* = 0.61
**SARS-CoV-2 variant**	
Delta/omicron	6/70 (7.9%/92.1%)
**At least one vaccination**	
No/yes	8/68 (10.5%/89.5%)
Two or more relevant comorbidities *	
No/yes	29/47 (38.2%/61.8%)
**Smoking**	
**No/yes**	56/20 (73.7%/26.3%)
**Breathing support**	
No/yes	61/15 (80.3%/19.7%)
COVID-19 pneumonia **	
No/yes	64/12 (84.2%/15.8%)
**Systemic treatment**	
No/yes	42/34 (55.3%/44.7%)
**Length of in-patient treatment**	
Median (range)	7.5 days (1–78)
**WHO clinical progression scale**	
Mild/moderate	
I	54 (71.1%)
II	13 (17.1%)
Severe/death	
III	6 (7.9%)
IV	3 (3.9%)

* Lung diseases, cardiovascular diseases, diabetes, and obesity. ** Based on X-ray or computed tomography.

**Table 2:** A comprehensive overview of the tubes utilized in flow cytometry, along with a detailed description of the fluorochrome mouse anti-human monoclonal antibodies employed.

**Tube 1 Exhausted T Cells**
CD45-PerCp-Cy5-5
CD3-BV605
CD4-BV786
CD8-BUV395
PD1(CD279)-BUV737
CD39-PE-594
TIM3(CD366)-BB515
TIGIT-BV421
**Tube 2 Exhausted NK Cells**
CD45-PerCp-Cy5-5
CD56-BV786
CD16-BUV395
PD1(CD279)-BUV737
LAG3(CD223)-PE-CF-594
TIM3(CD366)-BB515
TIGIT-BV421
**Tube 3 Monocytes**
CD45-PerCp-Cy5-5
CD11-BV605
CD14-CF594
CD16-BUV395
CD66a-BV786
CD200R1-BV421

**Table 3:** Flow cytometry data of peripheral blood mononuclear cells in healthy controls and patients with COVID-19. * = statistically significant.

Cell Surface Markers	Controls	COVID-19	*p*-Value
CD3+CD45+	69.3 (49.1–78.9)	70.1 (0.9–93.7)	=0.78
CD3+CD4+CD45+	64.2 (1.3–89.4)	69.5 (0.6–96)	=0.25
CD8+CD3+CD45+	26.4 (14.8–79.1)	19.1 (3.1–66)	=0.011 *
CD56+CD45+	89.8 (77.7–93.6)	65.5 (17.8–99)	<0.0001 *
CD16+CD45+	25.7 (12.6–37.9)	21.7 (0.77–51.3)	=0.85
CD56+CD16+CD45+	96.3 (89.5–98.6)	89.1 (58.2–98.1)	<0.0001 *
CD39+CD3+CD45+	1.7 (0.57–6.4)	5.9 (0.08–40.1)	=0.0004 *
CD39+CD4+CD3+CD45+	2.5 (0.19–26.1)	5.8 (0–41.3)	=0.016 *
CD39+CD8+CD3+CD45+	0.82 (0.03–6.2)	2.3 (0–100)	=0.0089 *

**Table 4:** Flow cytometry data of peripheral blood cells with immune checkpoint molecule co-expression (PD1, TIM3, and LAG3) in healthy controls and patients with COVID-19. * = statistically significant.

Cell Surface Markers	Controls	COVID-19	*p*-Value
PD1+CD16+CD45+	9.8 (2.1–24.8)	59.7 (29.3–94.7)	<0.0001 *
PD1+CD56+CD45+	9.1 (0.72–28.3)	62.4 (35.9–94.4)	<0.0001 *
PD1+CD3+CD45+	20.8 (8.4–58.6)	71.1 (40.1–96.7)	<0.0001 *
PD1+CD4+CD3+CD45+	17.2 (7.5–71.7)	72.3 (38.1–98.5)	<0.0001 *
PD1+CD8+CD3+CD45+	27.5 (8.6–58.3)	76.4 (0.6–98.2)	<0.0001 *
PD1+CD39+CD4+CD3+CD45+	30.7 (15.9–57.9)	69.1 (0–99.4)	<0.0001 *
PD1+CD39+CD8+CD3+CD45+	37.3 (0–100)	74.5 (0–100)	=0.0002 *
TIM3+CD16+CD45+	50.9 (31.5–73.1)	66.6 (11.7–91.2)	=0.0066 *
TIM3+CD56+CD45+	28.6 (12.1–45.8)	41.9 (11.3–67.9)	=0.0021 *
TIM3+CD3+CD45+	16.3 (4.9–32.3)	28.2 (13.4–82.8)	=0.0001 *
TIM3+CD4+CD3+CD45+	17 (1.1–25.7)	15.3 (4.4–84.9)	=0.64
TIM3+CD8+CD3+CD45+	19.8 (1.1–32.4)	21.9 (3.6–86,8)	=0.41
TIM3+CD39+CD4+CD3+CD45+	27 (5.3–51.5)	14.9 (0–80.7)	=0.0088 *
TIM3+CD39+CD8+CD3+CD45+	45.3 (15.6–57.1)	25.8 (0–96.7)	=0.060
LAG3+CD16+CD45+	2.5 (0.9–16.8)	16.8 (2.1–59.6)	<0.0001 *
LAG3+CD56+CD45+	2.7 (0.54–13.9)	10.9 (0.41–51.8)	=0.0004 *

**Table 6:** Univariable analysis including receiver operating curves (ROCs) and Chi^2^ tests in order to determine significant prognostic biomarkers for the outcomes of patients with COVID-19 (only significant data shown). In the multivariable regression analysis, we exclusively included dichotomized parameters revealing a significant area under the curve (AUC) ≥ 0.70 on ROC analysis or Chi^2^ test. OR = odds ratio; CI = confidence interval.

**Parameter**	**Univariable Analysis** WHO clinical progression scale (class III and IV)	**Multivariable Analysis** WHO clinical progression scale (class III and IV)
**LDH**	AUC 0.79, *p* < 0.0001 Criterion: >252, Youden index: 0.41	Did not remain in the model
**C-reactive protein**	AUC 0.90, *p* < 0.0001 Criterion: >39.8, Youden index: 0.78	Did not remain in the model
**Age**	AUC 0.74, *p* = 0.0009 Criterion: >63, Youden index: 0.41	Did not remain in the model
**CD39+CD45+**	AUC 0.71, *p* = 0.041 Criterion: >30.4, Youden index: 0.41	OR 51.4, 95% CI 1.5 to 1763 *p* = 0.029
**TIM3+CD39+CD4+CD3+CD45+**	AUC 0.73, *p* = 0.024 Criterion: >18.8, Youden index: 0.54	OR 22.6, 95% CI 1.8 to 277 *p* = 0.015
**TIM3+CD39+CD8+CD3+CD45+**	AUC 0.73, *p* = 0.020 Criterion: >39, Youden index: 0.47	Did not remain in the model

**Table 5:** Flow cytometry data of peripheral blood cells with immune checkpoint molecule co-expression (TIGIT and CD200R) in healthy controls and patients with COVID-19. * = statistically significant.

Cell Surface Markers	Controls	COVID-19	*p*-Value
TIGIT+CD16+CD45+	38.1 (13.5–68.7)	73.4 (13.2–96.1)	<0.0001
TIGIT+CD56+CD45+	26 (16.1–55)	48.1 (5.3–84)	=0.0009 *
TIGIT+CD3+CD45+	68.9 (3.8–91.9)	83 (19.2–97.9)	=0.10
TIGIT+CD4+CD3+CD45+	94.3 (19.1–99.6)	93.3 (10.6–99.7)	=0.47
TIGIT+CD8+CD3+CD45+	50.7 (12–88)	49 (11.5–90.8)	=0.95
TIGIT+CD39+CD4+CD3+CD45+	99.6 (67–100)	92.1 (0–100)	=0.0017 *
TIGIT+CD39+CD8+CD3+CD45+	68.4 (43.8–100)	75 (0–100)	=0.30
CD200R+CD16+CD45+	1.9 (0.9–19.3)	14.4 (0.45–90.8)	<0.0001 *
CD200R+CD11c+CD45+	12.2 (2.8–34)	30.3 (1.4–93.7)	=0.0005 *
CD200R+CD14+CD45+	20 (3.1–48.2)	44.2 (0–92.3)	=0.0003 *
CD200R+CD66+CD45+	19.5 (2.5–48.2)	67.2 (1.1–95.2)	<0.0001 *

## Data Availability

Derived data supporting the findings of this study are available from the corresponding author T.G. on reasonable request.

## Supplementary Materials

The following supporting information can be downloaded at https://www.mdpi.com/article/10.3390/v16050810/s1. Figure S1: The expression of T helper cells and cytotoxic T cells in patients with SARS-CoV-2 infection compared with healthy subjects; Figure S2: The expression of CD39 and ICPMs on T helper cells; Figure S3: The expression of CD39 and ICPMs on cytotoxic T cells; Figure S4: The expression of ICPMs on CD39+ T helper cells; Figure S5: The expression of ICPMs on CD39+ cytotoxic T cells; Figure S6: CD200R1 expression on NK cells, monocytes, and macrophages.
