# Supplementary material for: A Prospective Study Investigating Immune Checkpoint Molecule and CD39 Expression on Peripheral Blood Cells for the Prognostication of COVID-19 Severity and Mortality
Source: Viruses. 2024 May 20;16(5):810. doi: 10.3390/v16050810 (PMC11125582; doi:10.3390/v16050810)
Supplement: Supplementary file 1 [file viruses-16-00810-s001.zip › Neuer Ordner mit Objekten/Annotations Supplemental Material Figure A1-A6.docx]

Annotations Supplemental Material Figure A1-A6

**Suppl_FigA1:** The expression of T helper cells (CD4+ T cells) and cytotoxic T cells (CD8+ T cells) in patients with SARS-Cov-2 infection (A) compared to healthy subjects (B).

**Suppl_FigA2:** The expression of CD39 and PD-1 on T helper cells (CD4+ T cells) of subjects infected with SARS-CoV2 is significantly higher than in healthy individuals (p = 0.016 and p < 0.001). Conversely, TIM-3 and TIGIT expression did not show statistical significance (p = 0.64 and p = 0.47). A: The COVID group; B: The healthy control group.

**Suppl_FigA3:** The expression of CD39 and PD-1 on cytotoxic T cells of subjects infected with SARS-CoV2 is significantly higher than in healthy individuals (p = 0.004 and p < 0.001). Conversely, TIM-3 and TIGIT expression did not show any statistical significance (p = 0.41 and p = 0.95). A: The COVID group B: The helathy control group.

**Suppl_FigA4:** The expression of immune checkpoint molecules on CD39+ T helper cells of subjects infected with SARS-CoV2 is significantly higher than in healthy individuals (PD-1: p < 0.0001; TIGIT: p < 0.0088). The Coexpression of TIM-3+/CD39+ was significantly reduced in the infected (p = 0.0017). A: The COVID group B: The healthy control group.

**Suppl_FigA5:** The expression PD-1 on CD39+ cytotoxic T cells of subjects infected with SARS-CoV2 is significantly higher than in healthy individuals (p < 0.0002). Conversely, TIM-3 and TIGIT expression did not show statistical significance (p = 0.060 and p = 0.30). A: The COVID group B: The healthy control group.

**Suppl_FigA6:** The levels of CD200R1 expression show a significant increase in samples from SARS-Cov2-infected patients on NK cells, monocytes and macrophages, after determinizing the levels of CD11, CD14, CD16 and CD66a. A: The COVID group B: The healthy control group.
