# Supplementary figures and images for: A Prospective Study Investigating Immune Checkpoint Molecule and CD39 Expression on Peripheral Blood Cells for the Prognostication of COVID-19 Severity and Mortality
Source: Viruses. 2024 May 20;16(5):810. doi: 10.3390/v16050810 (PMC11125582; doi:10.3390/v16050810)

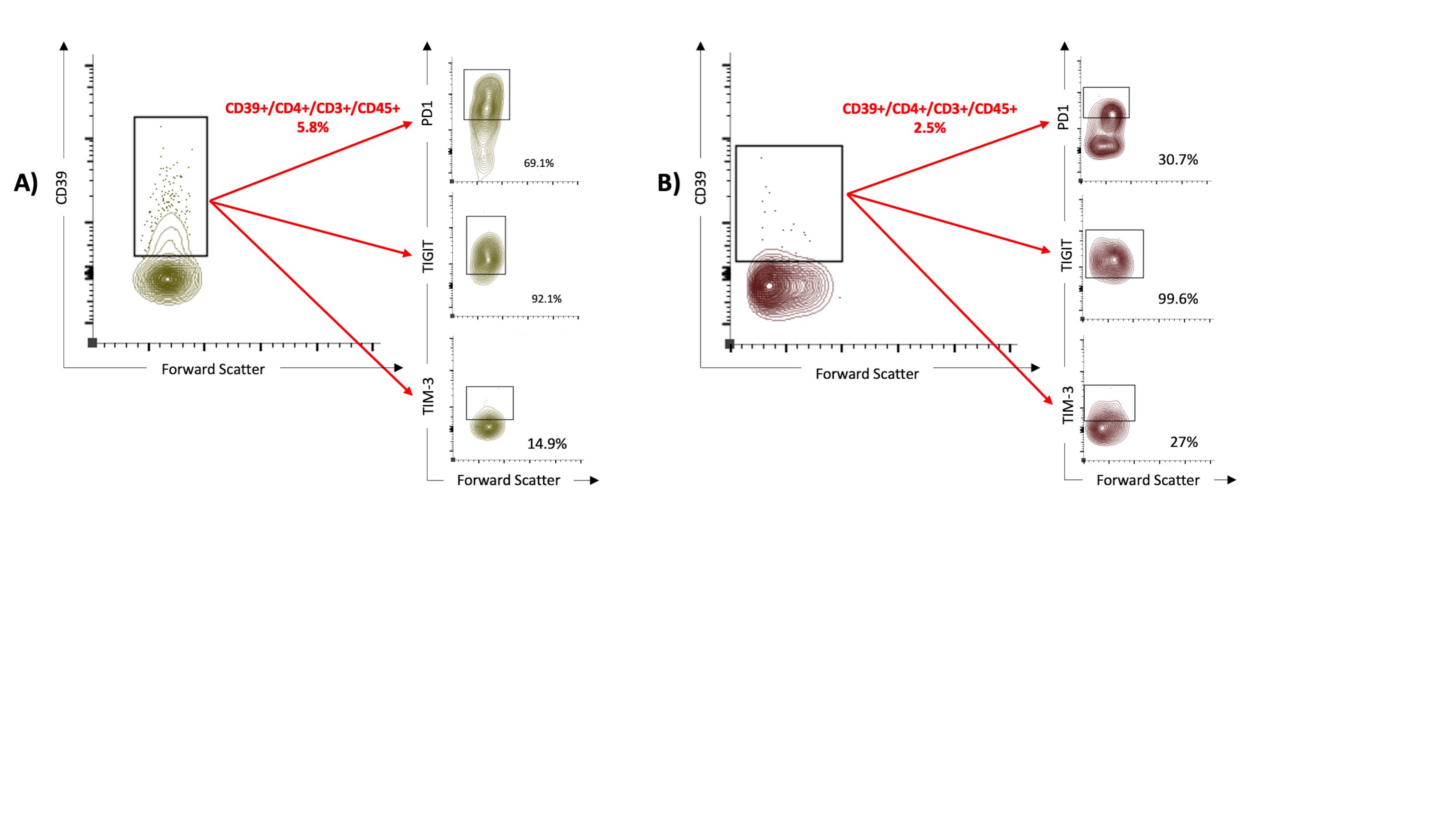

Supplement: Supplementary file 1 [file viruses-16-00810-s001.zip › Neuer Ordner mit Objekten/Suppl_FigA4.jpg]

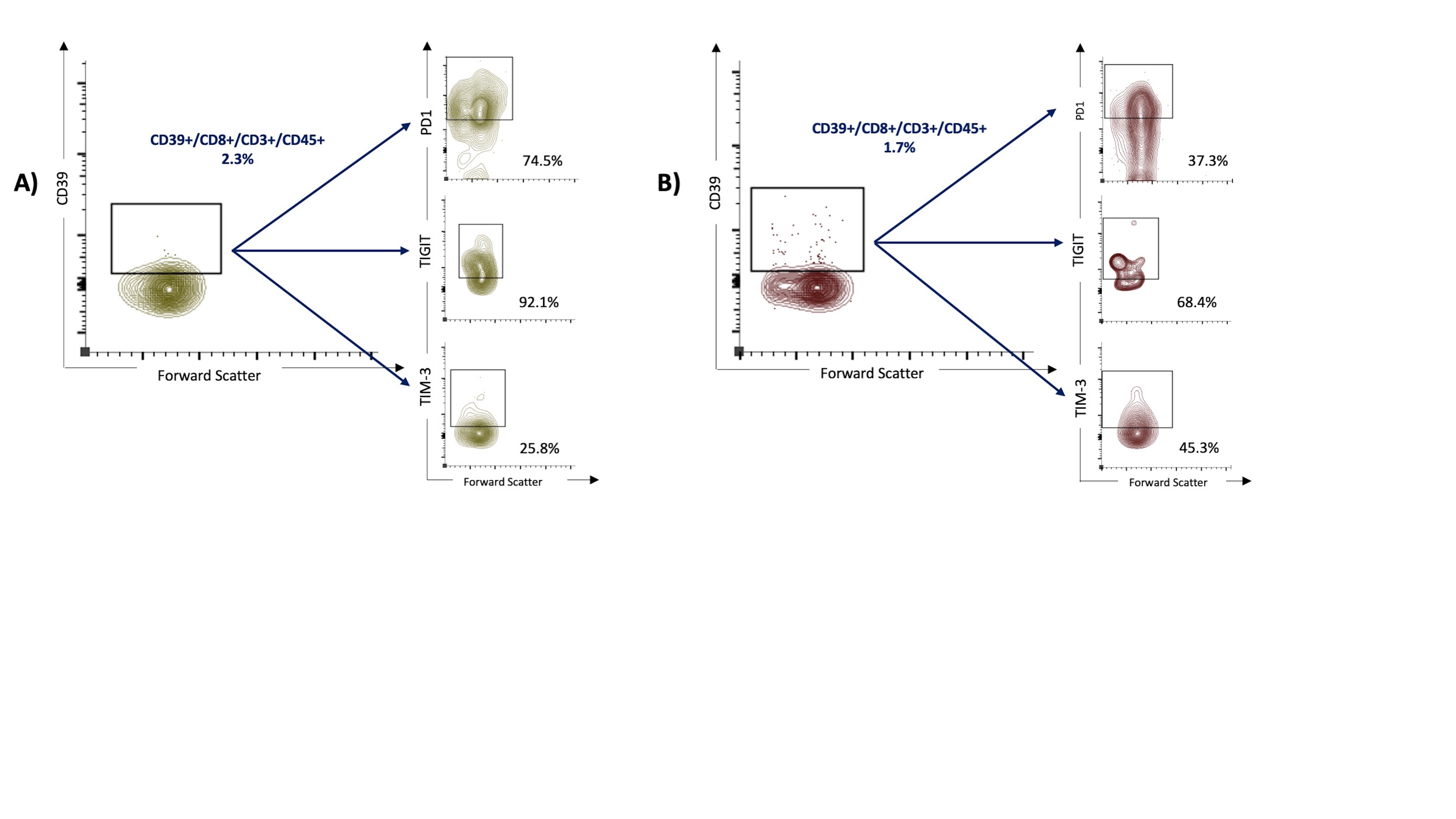

Supplement: Supplementary file 1 [file viruses-16-00810-s001.zip › Neuer Ordner mit Objekten/Suppl_FigA5.jpeg]

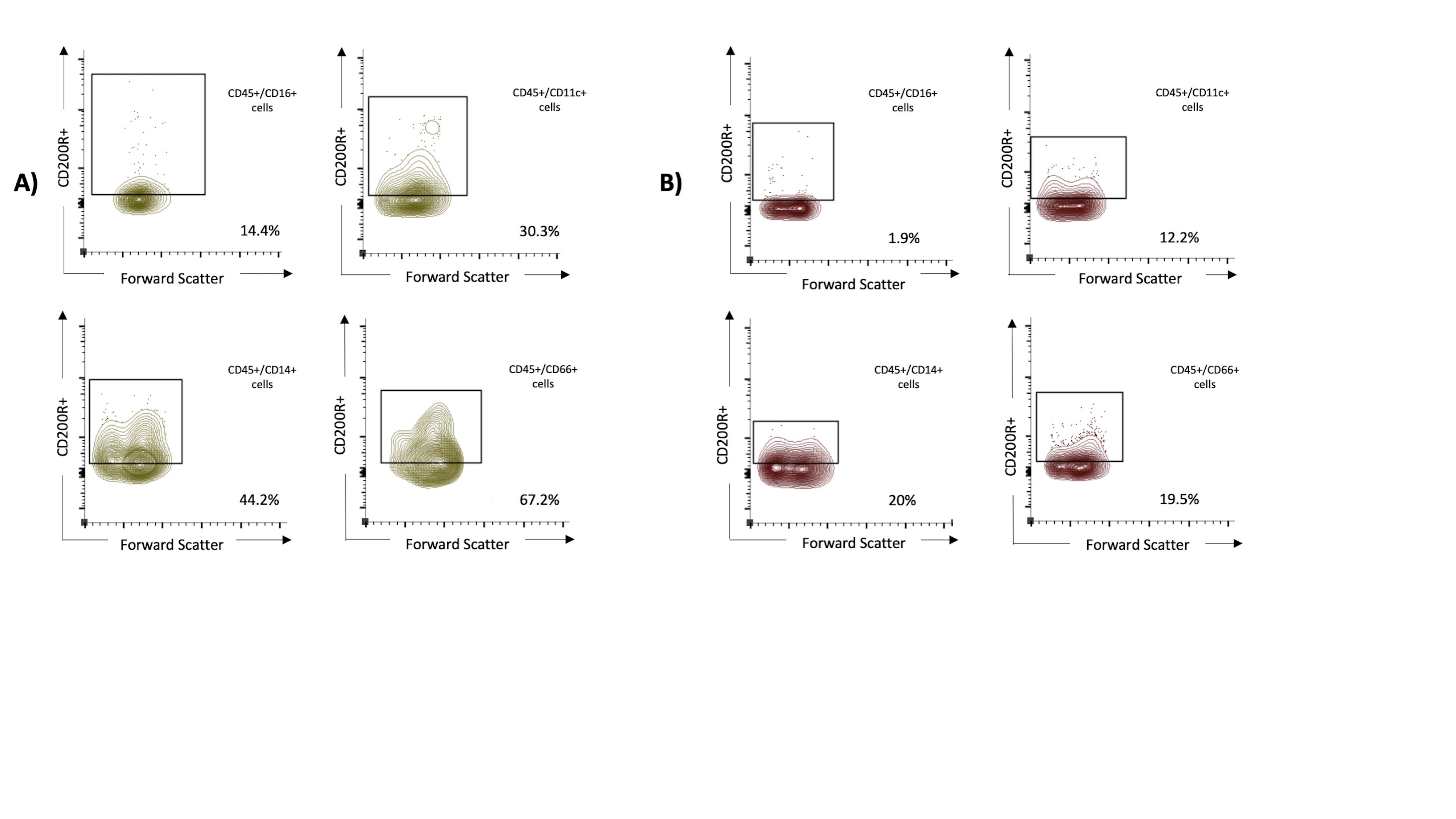

Supplement: Supplementary file 1 [file viruses-16-00810-s001.zip › Neuer Ordner mit Objekten/Suppl_FigA6.jpg]

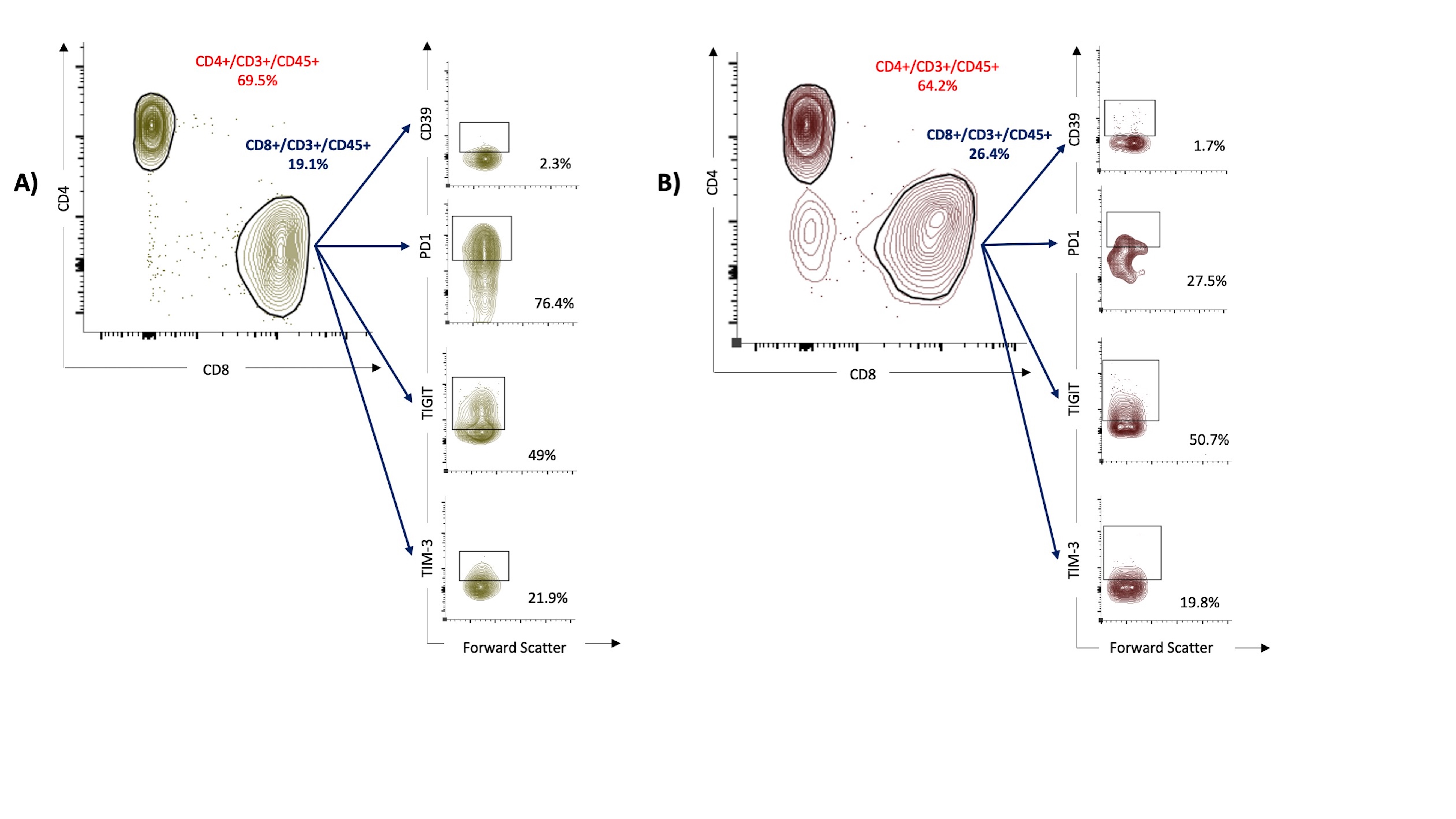

Supplement: Supplementary file 1 [file viruses-16-00810-s001.zip › Neuer Ordner mit Objekten/Suppl_FigA3.jpg]

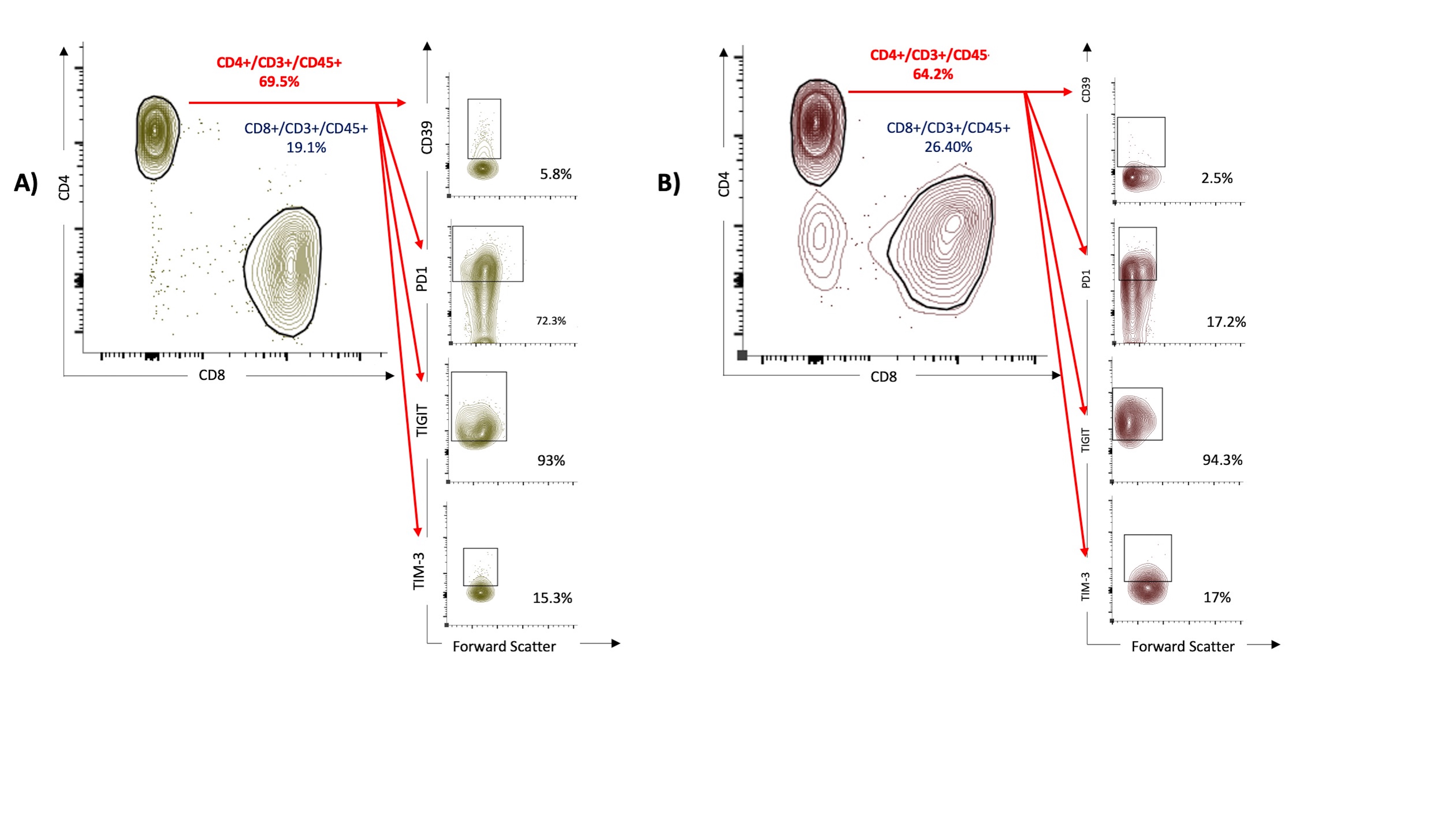

Supplement: Supplementary file 1 [file viruses-16-00810-s001.zip › Neuer Ordner mit Objekten/Suppl_FigA2.jpeg]

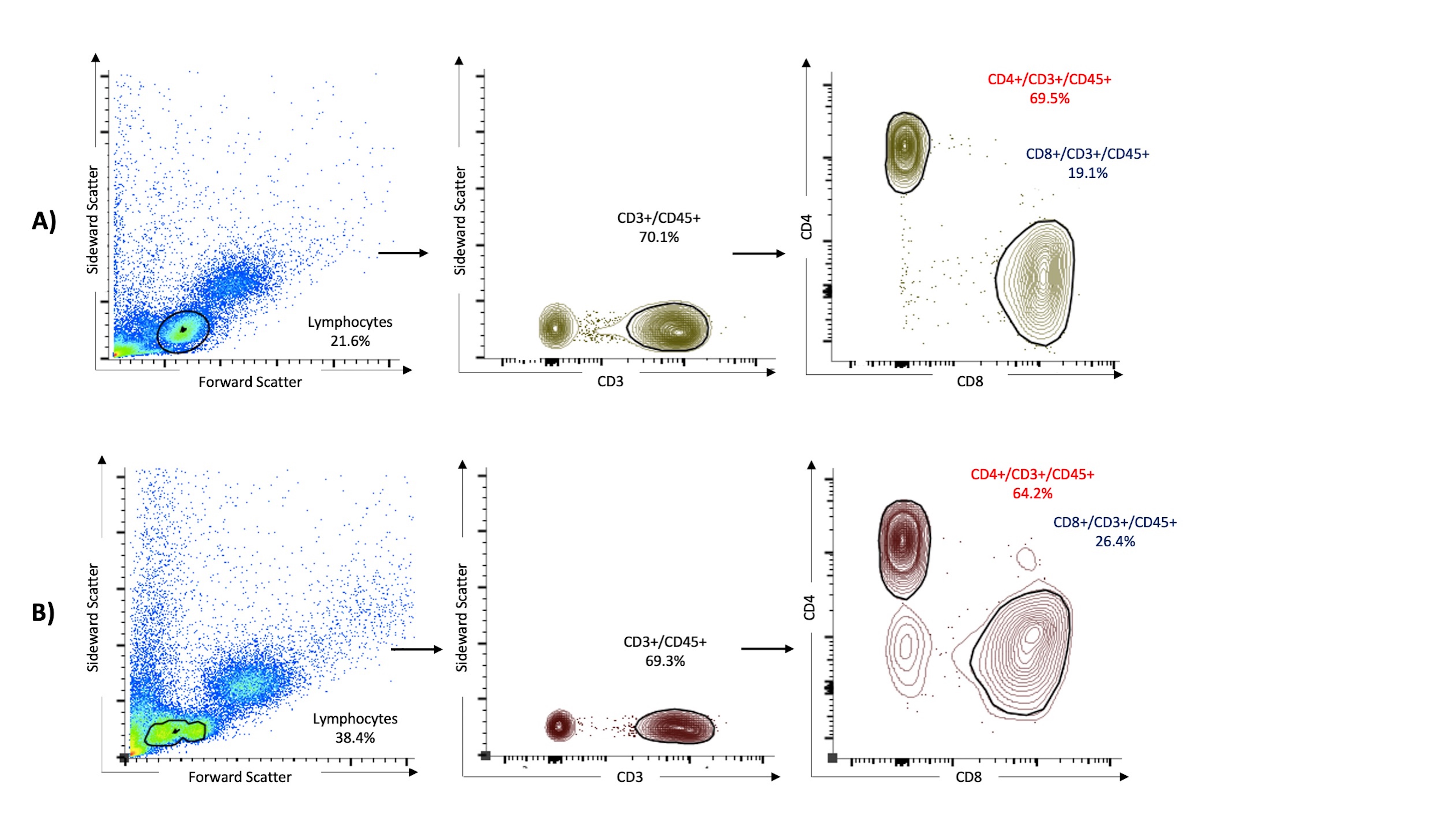

Supplement: Supplementary file 1 [file viruses-16-00810-s001.zip › Neuer Ordner mit Objekten/Suppl_FigA1.jpeg]
